# Limonene through Attenuation of Neuroinflammation and Nitrite Level Exerts Antidepressant-Like Effect on Mouse Model of Maternal Separation Stress

**DOI:** 10.1155/2021/8817309

**Published:** 2021-01-29

**Authors:** Zahra Lorigooini, Shakiba Nasiri Boroujeni, Mohammad Sayyadi-Shahraki, Mohammad Rahimi-Madiseh, Elham Bijad, Hossein Amini-khoei

**Affiliations:** Medical Plants Research Center, Basic Health Sciences Institute, Shahrekord University of Medical Sciences, Shahrekord, Iran

## Abstract

**Methods:**

Mice were randomly divided into experimental groups as follows: the control group received normal saline and MS groups received normal saline, limonene (10 and 20 mg/kg), L-NAME (10 mg/kg), L-arginine (L-arg) (75 mg/kg), limonene (10 mg/kg) plus L-NAME, and limonene (20 mg/kg) plus L-arg. Behavioral tests including the forced swimming test (FST), open field test (OFT), and splash test were performed. Finally, serum and hippocampal nitrite levels as well as the expression of inflammatory genes (IL-1*β* and TNF-*α*) in the hippocampus were measured.

**Results:**

We showed that MS caused depressive-like behavior. Treatment of MS mice with limonene reduced the duration of immobility time in FST and increases the grooming activity time in the splash test. Limonene also reduces serum and brain nitrite levels and reduces the expression of IL-1*β* and TNF-*α* in the hippocampus. We found that L-NAME potentiated the effects of a subeffective dose of limonene.

**Conclusion:**

We concluded that the antidepressant-like effects of limonene are probably mediated through inhibition of neuroinflammation and attenuation of nitrite levels in the hippocampus.

## 1. Introduction

Depression is one of the most common psychiatric disorders that is a common and important cause of disability in the world [1, 2]. According to the World Health Organization, about 264 million people worldwide suffer from depression [3]. Maternal separation (MS) is an approved model designed to induce stress during the early life in rodents [4]. MS is defined as the lack of care, short-term care, or repeated separation from mothers during early life, which undesirably affect the development of the brain. Infants under MS stress are prone to development of anxiety, depression, memory loss, and neurological disorders in adulthood [4].

It has been determined that there is an interrelationship between neural inflammation and depression. Evidence suggests that some inflammatory mediators, such as tumor necrosis factor alpha (TNF-*α*) and interleukin-1beta (IL-1*β*), are involved in the development of depressive disorders [5, 6]. Furthermore, previous studies have shown that oxidative and nitrosative stresses usually play a key role in the pathophysiology of depression by activating the immune response [7]. In this regard, it has been well determined that nitrite oxide (NO) acts as an activator of neuroinflammatory response [8]. Studies have also shown that the levels of inflammatory markers are increased in people with depressive symptoms [9]. It seems that the association between inflammatory reactions with depression led to a lack of full response to current antidepressants which commonly affect serotonergic neurotransmission [10]. Considering the fact of the growing prevalence of depression, there is a demanding need to introduce new effective agents with favorable and potent pharmacological possessions as well as low side effects.

Limonene is one of the most common terpenes (C_10_H_16_) in nature and is a major component of citrus extracts such as lemon, orange, tangerine, and grapefruit [11]. It has been shown that D-limonene reduced oxidative stress and inflammatory responses [12, 13]. Evidences have demonstrated that limonene through the effect on the parasympathetic system and central neurotransmitter activity possessed antidepressant effect [14]. However, the exact and complete mechanisms involved in the antidepressant effect of limonene have not yet been fully identified.

Considering the role of neuroinflammation and NO in the pathophysiology of depression as well as the reported neuroprotective effects for limonene, this study was performed to evaluate the possible reducing effects of hippocampal neuroinflammation and nitrite level on the antidepressant-like effect of limonene in a mouse model of MS stress.

## 2. Material and Methods

### 2.1. Ethical Approval

All procedures were carried out in accordance with the regulations of the University and the *Guide for the Care and Use of Laboratory Animals* of the National Institutes of Health (Ethics code: IR.SKUMS.REC.1397.129) and *Guide for the Care and Use of Laboratory Animals* (8th edition, National Academies Press). Full efforts were made to reduce the use of animals and to advance their welfare.

### 2.2. Maternal Separation

Pregnant NMRI mice (first-day pregnancies) were purchased from the Pasteur laboratory (Pasteur Institute, Tehran, Iran) and kept under standard laboratory conditions (12-hour periods of darkness and light and at 22 ± 2°C and free access to water and food). The birthday was considered the postnatal day (PND) = 0. Pups in PND = 2 up to PND = 14 were separated from their mothers for 3 constant hours daily [15, 16]. At the end of PND = 14, the pups returned to their mother's cages and were kept intact until day 21. From day 21, mice were isolated from their mother and were kept in cages in groups of 4 until PND 60 (22–30 g weight). Control mice were kept in the mother cage from PND 0 to PND 21 without manipulation and were then kept in cages in groups of 4 from PND 21 to PND 60.

### 2.3. Drugs

The drugs used in this study were as follows: (1) limonene, (2) L-NAME (a nonselective nitric oxide synthase or NOS inhibitor), and (3) L-arginine (a NO precursor). All agents were bought from Sigma, St. Louis, MO, USA. Drugs were dissolved in 0.9% physiological saline and injected as a single dose via the intraperitoneal route (i.p.) with a volume of 5 ml/kg body weight.

### 2.4. Animals and Study Design

**Figure 1:**
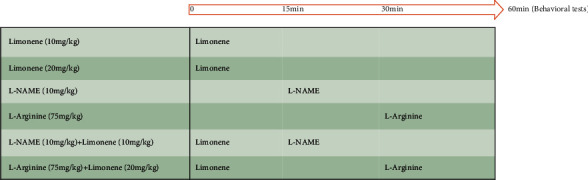
Schematic of the study design.

64 male NMRI mice were randomly divided into 8 groups (*n* = 8). Group 1 was the control mice which received normal saline. Groups 2-8 were MS mice which received normal saline, limonene at doses of 10 and 20 mg/kg, L-NAME at a dose of 10 mg/kg, L-arginine (l-arg) at a dose of 75 mg/kg, an ineffective dose of limonene plus L-NAME, and an effective dose of limonene plus L-arginine, respectively. Mice were treated with L-arg (30 min), L-NAME (45 min), and limonene (60 min) prior to the behavioral tests. The dose and time of drug administrations were chosen according to the previous studies [17–19] as well as our pilot studies. Each mouse was used only for one test (the same mice were used for the FST and the OFT, and different sets of mice were used for the splash test). In order to prevent the effects of manipulation by different experimenters on animals, all experiments were conducted by a single experimenter. Each experimental group contained 8 mice for behavioral tests and 4 samples for molecular assessments (gene expression and nitrite level). The schematic of the study design is shown in Figure 1.

### 2.5. Forced Swimming Test (FST)

In this experiment, the immobility time of the mice was recorded as a reflection of depressive-like behavior. To do this, a glass container (25 × 12 × 15 cm) filled with 25°C water and mouse was placed gently in the water from a height of 20 cm. Disruption of mouse movements was considered immobility. The test time was 6 minutes; the first 2 minutes was considered to match the animal with the current conditions, and the immobility time was measured for the next 4 minutes [20].

### 2.6. Open Field Test (OFT)

The OFT was performed to evaluate the locomotor activity following treatments. The OFT was done immediately before the FST to consider ambulatory behavior as well as to confirm that adjustments which occur in motor activity did not affect the immobility time in the FST. The OFT device is a white Plexiglas with dimensions of 30 × 50 × 50 cm. Each mouse was gently placed in the middle of the device. Its movements were recorded by a camera for 5 minutes and evaluated by Ethovision software version 8. In the OFT, the horizontal (distance moved, number of crossing by 4 feet from each square) and vertical (number of rearings) activities were measured [21]. The apparatus was cleaned with 70% ethanol after the experiment with each mouse.

### 2.7. Splash Test

The splash test was used to examine personal care and motivational problems in mice. To do this, a 10% sucrose solution was sprayed on the dorsal coat of the mouse, and its behavior was filmed for 5 minutes. In this test, self-cleaning activities including nose/face cleaning, head washing, and body grooming were measured [22].

### 2.8. Nitrite Assay

The nitrite level as a stable NO product was measured in the hippocampus and serum samples. In brief, mice were sacrificed under anesthesia using diethyl ether, and the hippocampus was dissected on the ice-cold surface and directly placed into liquid nitrogen. Hippocampus homogenates were prepared, and nitrite concentrations were measured using a colorimetric assay based on the Griess reaction [23]. Briefly, each well was loaded with 100 *μ*l samples and mixed with 100 *μ*l Griess reagent. The absorbance was measured at 540 nm in an automated plate reader, after ten-minute incubation at room temperature. The level of nitrite was determined by reference to a standard curve of sodium nitrite (Sigma, USA) and normalized to the weight of each sample.

### 2.9. The Expression of Inflammatory Genes

At the end of the experiment, the mice were sacrificed under anesthesia using diethyl ether, and the hippocampus was removed, and the expression of IL-1*β* and TNF-*α* genes was assessed by real-time PCR. The reaction for each gene was tripled and repeated twice. The required specific primers were designed using Primer 3 software version 0.4.0, and the H2afz gene was considered a normalizer; the rate of change in expression of the desired genes was compared with the control group. Finally, the data were calculated using the PFAFFL formula. The primer sequences are listed in Table 1.

### 2.10. Data Analysis

The data were analyzed using SPSS software. One-way ANOVA followed by Tukey's post hoc test was used to determine significant differences among groups. Data were recorded as the mean ± S.E.M., and *P* < 0.05 was considered statistically significant.

## 3. Results

### 3.1. Immobility Time in the FST

**Figure 2:**
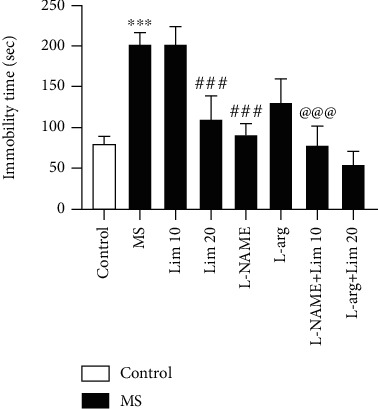
Duration of immobility in the forced swimming test. Values are presented as the mean ± S.E.M. from 8 animals and were analyzed by one-way ANOVA followed by Tukey's post hoc test. ^∗∗∗^*P* < 0.001 compared to the control group. ^###^*P* < 0.001 compared to the MS group. ^@@@^*P* < 0.001 compared to the MS group receiving limonene at a dose of 10 mg/kg. MS: maternal separation; Lim: limonene; L-arg: L-arginine.

The results showed that MS significantly increased the immobility time in the FST (*P* < 0.001, Figure 2). The immobility time in the MS groups that received limonene at a dose of 20 mg/kg and L-NAME at a dose of 10 mg/kg significantly lowered than that of the saline-treated MS group (*P* < 0.001). Concomitant administration of limonene plus L-NAME to the MS mice significantly reduced the duration of immobility compared to the counterpart receiving a subeffective dose of limonene (10 mg/kg) alone (*P* < 0.001).

### 3.2. Locomotor Activity in the OFT

**Figure 3:**
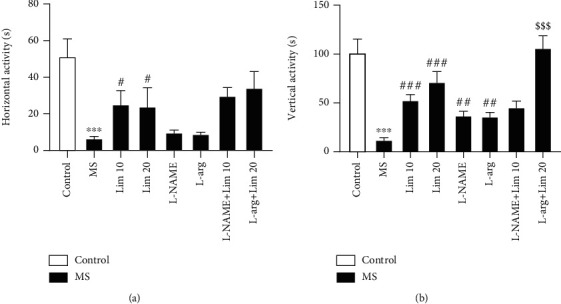
The locomotor activity in the OFT. (a) Horizontal activity and (b) vertical activity. Values are presented as the mean ± S.E.M. from 8 animals and were analyzed by one-way ANOVA followed by Tukey's post hoc test. ^∗∗∗^*P* < 0.001 compared to the control group. ^#^*P* < 0.05, ^##^*P* < 0.01, and ^###^*P* < 0.001 compared to the MS group. ^$$$^*P* < 0.001 compared to the group that received limonene at a dose of 20 mg/kg. MS: maternal separation; Lim: limonene; L-arg: L-arginine.

The results showed that the horizontal activity (Figure 3(a)) in the MS group was significantly lower than that of the control group (*P* < 0.001). The administration of limonene at doses of 10 and 20 mg/kg significantly increased the horizontal activity compared to the saline-treated MS group (*P* < 0.05).

Also, according to Figure 3(b), the vertical activity in the MS group is significantly lower than that of the control group (*P* < 0.001). Administration of limonene at doses of 10 and 20 mg/kg significantly increased vertical activity compared to the saline-treated counterpart (*P* < 0.001). Furthermore, injection of L-NAME and L-arg significantly increased the vertical activity compared to the MS counterpart (*P* < 0.01). Coadministration of limonene at the effective dose (20 mg/kg) plus L-arg significantly increased the vertical activity compared to the group that received the effective dose of limonene alone (*P* < 0.001).

### 3.3. Grooming Activity Time in the Splash Test

**Figure 4:**
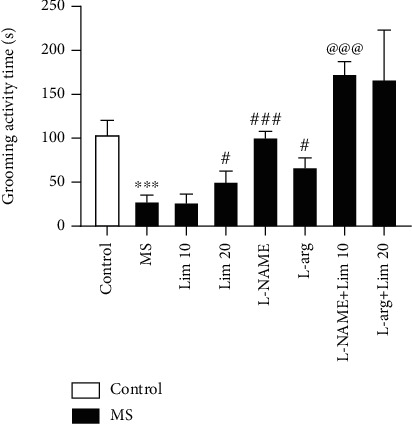
Grooming activity duration in the splash test. Values are presented as the mean ± S.E.M. from 8 animals and were analyzed by one-way ANOVA followed by Tukey's post hoc test. ^∗∗∗^*P* < 0.001 compared to the control group. ^#^*P* <0.05 and ^###^*P* < 0.001 compared to the MS group. ^@@@^*P* < 0.001 compared to the group that received limonene at a dose of 10 mg/kg. MS: maternal separation; Lim: limonene; L-arg: L-arginine.

Based on the results shown in Figure 4, the duration of grooming activity in the MS group is significantly lower than that of the control group (*P* < 0.001). Administration of limonene at a dose of 20 mg/kg significantly increased grooming activity compared to the saline-treated MS group (*P* < 0.05). Also, administration of L-arg to the MS mice significantly increased the duration of grooming activity compared to the saline-treated MS group (*P* < 0.05). We found that L-NAME significantly increased the grooming activity time compared to the saline-treated MS group (*P* < 0.001). Coadministration of L-NAME plus a subeffective dose of limonene (10 mg/kg) significantly increased the duration of grooming activity compared to the MS group that received a subeffective dose of limonene alone (*P* < 0.001).

### 3.4. The Brain and Serum Nitrite Levels

**Figure 5:**
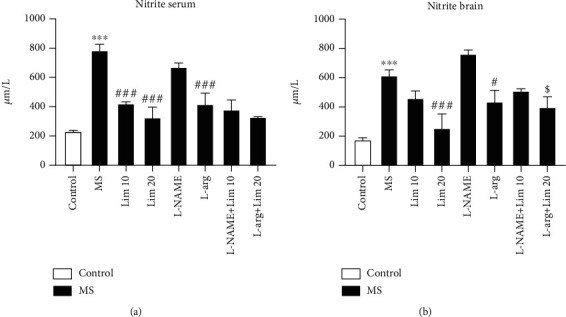
Nitrite levels in the brain and serum samples. Values are presented as the mean ± S.E.M. from 4 samples and were analyzed by one-way ANOVA followed by Tukey's post hoc test. ^∗∗∗^*P* < 0.001 compared to the control group. ^#^*P* < 0.05 and ^###^*P* < 0.001 compared to the MS group. ^$^*P* < 0.05 compared to the group that received limonene at a dose of 20 mg/kg. MS: maternal separation; Lim: limonene; L-arg: L-arginine.

Based on our results (Figure 5(a)), MS significantly increased the nitrite levels in the serum compared to the control group (*P* < 0.001). Administration of limonene at doses of 10 and 20 mg/kg significantly reduced the serum nitrite levels compared to the saline-treated MS group (*P* < 0.001). Also, administration of L-arg significantly reduced the serum nitrite levels compared to the MS group treated with saline (*P* < 0.001).

Figure 5(b) shows that MS significantly increased the brain nitrite levels compared to the control group (*P* < 0.001). The results showed that administration of limonene at a dose of 20 mg/kg as well as L-arg significantly reduced the brain nitrite levels compared to the saline-treated MS mice (*P* < 0.001 and *P* < 0.05, respectively). Coadministration of limonene at an effective dose of 20 mg/kg plus L-arg significantly increased the brain nitrite levels compared to the group that received an effective dose of limonene alone (*P* < 0.05).

### 3.5. The Expression Inflammatory Genes in the Hippocampus

**Figure 6:**
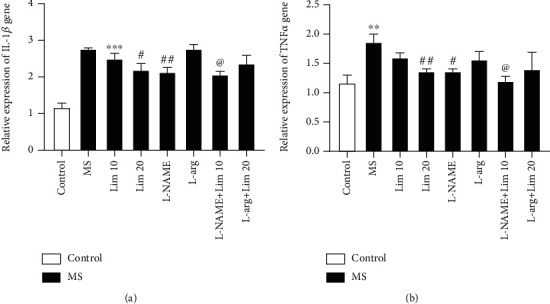
The expression of inflammatory genes in the hippocampus. (a) *Il-1β* and (b) *Tnf-α*. Values are presented as the mean ± S.E.M. from 4 samples and were analyzed by one-way ANOVA followed by Tukey's post hoc test. ^∗∗^*P* < 0.01 and ^∗∗∗^*P* < 0.001 compared to the control group. ^#^*P* < 0.05 and ^##^*P* < 0.01 compared to the MS group. ^@^*P* < 0.05 compared to the group that received limonene (10 mg/kg). MS: maternal separation; Lim: limonene; L-arg: L-arginine.

As shown in Figure 6(a), the expression of *Il-1β* in the hippocampus of MS mice significantly increased compared to the control group (*P* < 0.001). Administration of limonene at a dose of 20 mg/kg resulted in a significant reduction in *Il-1β* gene expression compared to the saline-treated MS group (*P* < 0.05). L-NAME significantly reduced the expression of *Il-1β* compared to the MS counterpart (*P* < 0.01). Coadministration of the subeffective dose of limonene (10 mg/kg) plus L-NAME significantly reduced the expression of *Il-1β* compared to the group that received the subeffective dose of limonene alone (*P* < 0.05).

The results (Figure 6(b)) showed that MS significantly increased the expression of *Tnf-α* gene compared to the control group (*P* < 0.01). Limonene at a dose of 20 mg/kg significantly decreased the expression of the *Tnf-α* gene compared to the MS counterpart (*P* < 0.01). Also, administration of L-NAME significantly reduced the expression of *Tnf-α* gene compared to the saline-treated MS group (*P* < 0.05). The results showed that coadministration of the subeffective dose of limonene (10 mg/kg) plus L-NAME significantly decreased the expression of *Tnf-α* compared to the group that received the subeffective dose of limonene alone (*P* < 0.05).

## 4. Discussion

Our findings showed that maternal separation caused depressive-like behavior in mice. Limonene exerted antidepressant-like effects on MS mice. We observed that MS led to an increase in nitrite levels as well as overexpression of genes relevant to neuroinflammation. The results showed that limonene reduced the neuroinflammatory response and also reduced nitrite levels in the hippocampus. Furthermore, coinjections of the subeffective dose of limonene with the subeffective dose of L-NAME significantly potentiated the antidepressant-like effect of the subeffective dose of limonene. In addition, coinjection of the effective dose of limonene with the effective dose of L-arg attenuated the antidepressant effect of the effective dose of limonene.

Marais et al. showed that the MS, by changing the levels of corticosteroids and neurotrophin in the hippocampus, increases the risk of depressive-like behaviors in the FST following chronic stress [24]. Ample evidence showed that MS increased the duration of immobility in the FST and reduced the grooming activity time in the splash test [25]. Tchenio et al. showed that MS has long-term effects on brain and behavior development and led to the development of depression in adulthood [26]. Our findings determined that the MS causes depressive-like behaviors in the FST and splash test in mice. In this way, the immobility time in MS mice in the FST test is increased and the time of self-care and self-cleaning is significantly reduced in the splash test. In addition, we performed the OFT to approve that locomotor activity subsequent to treatments does not affect the FST results, and the immobility of animals in the FST is not associated with their hypolocomotion [27]. In the other word, OFT was performed directly before the FST to measure ambulatory behavior and approve that inconsistencies which happen in motor activity did not affect the immobility time in the FST.

d'Alessio et al. showed that D-limonene has significant antistress and antiaggression effects [14]. Zhang et al. showed that inhaling limonene significantly reduced depressive-like behaviors. They demonstrated that alterations in neuroendocrine, neurotrophic, and monoaminergic systems have been involved in the antidepressant-like effects of limonene [28]. Our study, along with other studies, showed that treatment of MS mice with limonene significantly reduced the duration of immobility in the FST and increased the grooming activity time in the splash test indicating antidepressive-like properties of this agent.

Ludka et al. showed that drugs that reduce NO levels have possessed antidepressant effects on major depressive disorder [29]. Previously, it had been thought that antidepressants only affected neurotransmitters [30]. However, there are evidences showing that some commonly prescribed drugs may exert their effects via normalizing the level of NO [31]. About changes in NO levels in mice exposed to chronic stress, it has been determined that the plasma level of NO was clearly increased [32]. Our study, along with other studies, showed that MS increased nitrite levels in serum and the brain. Also, treatment of MS mice with limonene significantly reduced brain and serum nitrite levels. This finding suggests that this mechanism is at least partially involved in the antidepressant effect of limonene on MS mice.

In a study in 2019, it was found that people with severe symptoms of depression have high levels of preinflammatory cytokines [33]. It has been verified that the increasing levels of inflammatory factors such as IL-1, IL-6, and TNF-*α* are associated with depressive-like behaviors [34]. It has been shown that MS increased the expression of IL-1*β* and TNF-*α* genes in the hippocampus [35]. Studies have showed that some agents effectively mitigating depressive-like behaviors significantly reduced the expression of genes relevant to neuroimmune response in the hippocampus [36, 37]. Studies have shown that limonene reduced the inflammatory response and decreased the levels of inflammatory cytokines [14]. In our study, MS increased the expression of IL-1*β* and TNF-*α* inflammatory genes in the hippocampus, indicating that inflammatory response in the hippocampus was associated with depressive behaviors in MS mice. Also, limonene significantly reduced the expression of these inflammatory genes, indicating that this mechanism partially, at least, is involved in the antidepressant-like effect of limonene on MS mice.

In case of interventional study with NO precursor and NOS inhibitor, we showed that coadministration of L-NAME plus subeffective dose of limonene significantly potentiated the antidepressant-like effect of limonene at the subeffective dose. Furthermore, coadministration of the effective dose of L-arg with the effective dose of limonene significantly mitigated the antidepressant-like effect of limonene. Our results demonstrated that modulation of the nitrergic system, partially at least, is involved in the antidepressant-like effect of limonene on MS mice.

## 5. Conclusion

In conclusion, we found that limonene exerts antidepressant-like effects on MS mice by reducing hippocampal nitrite levels and the neuroimmune response. We found that the nitrergic system is involved, at least in part, in the antidepressant-like effect of limonene in MS mice, in which coadministration of L-NAME and L-arg enhances and decreases the effect of limonene, respectively.

## Figures and Tables

**Table 1:** Primer sequences used in PCR amplification.

Primer	Forward sequence	Reverse sequence
*H2afz*	TCATCGACACCTGAAATCTAGGA	AGGGGTGATACGCTTTACCTTTA
*Tnf-α*	CTGAACTTCGGGGTGATCGG	GGCTTGTCACTCGAATTTTGAGA
*Il-1β*	GAAATGCCACCTTTTGACAGTG	TGGATGCTCTCATCAGGACAG

## Data Availability

Our data are available during manuscript submission.

## References

[1] Cooney G. M., Dwan K., Greig C. A. (2013). Exercise for depression. *Cochrane Database Systematic Reviews*.

[2] Silva M. T., Galvao T. F., Martins S. S., Pereira M. G. (2014). Prevalence of depression morbidity among Brazilian adults: a systematic review and meta-analysis. *Brazilian Journal of Psychiatry*.

[3] Hoying J., Melnyk B. M., Hutson E., Tan A. (2020). Prevalence and correlates of depression, anxiety, stress, healthy beliefs, and lifestyle behaviors in first-year graduate health sciences students. *Worldviews on Evidence-Based Nursing*.

[4] Anjomshoa M., Boroujeni S. N., Ghasemi S. (2020). Rutin via increase in the CA3 diameter of the hippocampus exerted antidepressant-like effect in mouse model of maternal separation stress: possible involvement of NMDA receptors. *Behavioural Neurology*.

[5] Liu B., Xu C., Wu X. (2015). Icariin exerts an antidepressant effect in an unpredictable chronic mild stress model of depression in rats and is associated with the regulation of hippocampal neuroinflammation. *Neuroscience*.

[6] Dutcher J. P., Logan T., Gordon M. (2000). Phase II trial of interleukin 2, interferon alpha, and 5-fluorouracil in metastatic renal cell cancer: a cytokine working group study. *Clinical Cancer Research*.

[7] Leonard B., Maes M. (2012). Mechanistic explanations how cell-mediated immune activation, inflammation and oxidative and nitrosative stress pathways and their sequels and concomitants play a role in the pathophysiology of unipolar depression. *Neuroscience & Biobehavioral Reviews*.

[8] Beheshti F., Hashemzehi M., Hosseini M., Marefati N., Memarpour S. (2020). Inducible nitric oxide synthase plays a role in depression- and anxiety-like behaviors chronically induced by lipopolysaccharide in rats: Evidence from inflammation and oxidative stress. *Behavioural Brain Research*.

[9] Valkanova V., Ebmeier K. P., Allan C. L. (2013). CRP, IL-6 and depression: a systematic review and meta-analysis of longitudinal studies. *Journal of Affective disorders*.

[10] Miller A. H., Raison C. L. (2016). The role of inflammation in depression: from evolutionary imperative to modern treatment target. *Nature Reviews Immunology*.

[11] Yoo Z.-W., Kim N.-S., Lee D.-S. (2004). Comparative analyses of the flavors from Hallabong (Citrus sphaerocarpa) with lemon, orange and grapefruit by SPTE and HS-SPME combined with GC-MS. *Bulletin-Korean Chemical Society*.

[12] Manuele M. G., Barreiro Arcos M. L., Davicino R., Ferraro G., Cremaschi G., Anesini C. (2009). Limonene exerts antiproliferative effects and increases nitric oxide levels on a lymphoma cell line by dual mechanism of the ERK pathway: relationship with oxidative stress. *Cancer Investigation*.

[13] Chaudhary S., Siddiqui M., Athar M., Alam M. S. (2012). D-Limonene modulates inflammation, oxidative stress and Ras-ERK pathway to inhibit murine skin tumorigenesis. *Human & Experimental Toxicology*.

[14] d'Alessio P. A., Bisson J.-F., Béné M. C. (2014). Anti-stress effects of d-limonene and its metabolite perillyl alcohol. *Rejuvenation Research*.

[15] Desbonnet L., Garrett L., Clarke G., Kiely B., Cryan J. F., Dinan T. G. (2010). Effects of the probiotic *Bifidobacterium infantis* in the maternal separation model of depression. *Neuroscience*.

[16] Boroujeni S. N., Lorigooini Z., Boldaji F. R., Amini-Khoei H. (2020). Diosgenin via NMDA receptor exerted anxiolytic-like effect in maternally separated mice. *Current Pharmaceutical Design*.

[17] Piccinelli A. C., Santos J. A., Konkiewitz E. C. (2014). Antihyperalgesic and antidepressive actions of (R)-(+)-limonene, *α*-phellandrene, and essential oil from Schinus terebinthifolius fruits in a neuropathic pain model. *Nutritional Neuroscience*.

[18] Cunha M. P., Pazini F. L., Ludka F. K. (2015). The modulation of NMDA receptors and L-arginine/nitric oxide pathway is implicated in the anti-immobility effect of creatine in the tail suspension test. *Amino Acids*.

[19] Ostadhadi S., Kordjazy N., Haj-Mirzaian A., Ameli S., Akhlaghipour G., Dehpour A. (2016). Involvement of NO/cGMP pathway in the antidepressant-like effect of gabapentin in mouse forced swimming test. *Naunyn-Schmiedeberg's Archives of Pharmacology*.

[20] Kotagale N., Deshmukh R., Dixit M., Fating R., Umekar M., Taksande B. (2020). Agmatine ameliorates manifestation of depression-like behavior and hippocampal neuroinflammation in mouse model of Alzheimer's disease. *Brain Research Bulletin*.

[21] Kim D. H., Kwon H., Choi J. W. (2020). Roles of GABA_A_ receptor *α*5 subunit on locomotion and working memory in transient forebrain ischemia in mice. *Progress in Neuro-Psychopharmacology and Biological Psychiatry*.

[22] Bampi S. R., Casaril A. M., Fronza M. G. (2020). The selenocompound 1-methyl-3-(phenylselanyl)-1 *H* -indole attenuates depression-like behavior, oxidative stress, and neuroinflammation in streptozotocin-treated mice. *Brain Research Bulletin*.

[23] Granger D. L., Taintor R. R., Boockvar K. S., Hibbs J. B. (1996). Measurement of nitrate and nitrite in biological samples using nitrate reductase and Griess reaction. *Methods in Enzymology*.

[24] Marais L., van Rensburg S. J., van Zyl J. M., Stein D. J., Daniels W. M. (2008). Maternal separation of rat pups increases the risk of developing depressive- like behavior after subsequent chronic stress by altering corticosterone and neurotrophin levels in the hippocampus. *Neuroscience Research*.

[25] Lorigooini Z., Sadeghi Dehsahraei K., Bijad E., Habibian Dehkordi S., Amini-Khoei H. (2020). Trigonelline through the attenuation of oxidative stress exerts antidepressant-and anxiolytic-like effects in a mouse model of maternal separation stress. *Pharmacology*.

[26] Tchenio A., Lecca S., Valentinova K., Mameli M. (2017). Limiting habenular hyperactivity ameliorates maternal separation-driven depressive-like symptoms. *Nature Communications*.

[27] Anjomshoa M., Boroujeni S., Bagheri E., Lorigooini Z., Amini-Khoei H. (2020). Possible involvement of N-methyl-D-aspartate receptor (NMDA-R) in the antidepressant-like effect of trigonelline in male mice. *Current Pharmaceutical Design*.

[28] Zhang L.-L., Yang Z.-Y., Fan G., Ren J.-N., Yin K.-J., Pan S.-Y. (2019). Antidepressant-like effect of *Citrus sinensis* (L.) Osbeck essential oil and its main component limonene on mice. *Journal of Agricultural and Food Chemistry*.

[29] Ludka F. K., Zomkowski A. D., Cunha M. P. (2013). Acute atorvastatin treatment exerts antidepressant-like effect in mice via the l-arginine-nitric oxide-cyclic guanosine monophosphate pathway and increases BDNF levels. *European Neuropsychopharmacology*.

[30] Taylor C., Fricker A. D., Devi L. A., Gomes I. (2005). Mechanisms of action of antidepressants: from neurotransmitter systems to signaling pathways. *Cellular Signalling*.

[31] Kudlow P., Cha D., Carvalho A., McIntyre R. (2016). Nitric oxide and major depressive disorder: pathophysiology and treatment implications. *Current Molecular Medicine*.

[32] Gao S.-F., Lu Y.-R., Shi L.-G. (2014). Nitric oxide synthase and nitric oxide alterations in chronically stressed rats: a model for nitric oxide in major depressive disorder. *Psychoneuroendocrinology*.

[33] Chen M., Wu E., Heijnen C., Fagundes C. (2019). Abstract 3141 Association between depression and inflammation among bereaved adults. *Brain, Behavior, and Immunity*.

[34] Leonard B. E. (2007). Inflammation, depression and dementia: are they connected?. *Neurochemical Research*.

[35] Jolodar S. K., Bigdeli M., Moghaddam A. H. (2020). Hypericin ameliorates maternal separation-induced cognitive deficits and hippocampal inflammation in rats. *Mini Reviews in Medicinal Chemistry*.

[36] Ramirez K., Niraula A., Sheridan J. F. (2016). GABAergic modulation with classical benzodiazepines prevent stress-induced neuro-immune dysregulation and behavioral alterations. *Brain, Behavior, and Immunity*.

[37] Bower J. E., Kuhlman K. R., Haydon M. D., Boyle C. C., Radin A. (2019). Cultivating a healthy neuro-immune network: a health psychology approach. *Social and Personality Psychology Compass*.

